# Effect of Process Parameters on Tensile Mechanical Properties of 3D Printing Continuous Carbon Fiber-Reinforced PLA Composites

**DOI:** 10.3390/ma13173850

**Published:** 2020-08-31

**Authors:** Hao Dou, Yunyong Cheng, Wenguang Ye, Dinghua Zhang, Junjie Li, Zhoujun Miao, Stephan Rudykh

**Affiliations:** 1 School of Mechanical Engineering, Northwestern Polytechnical University, Xi’an 710072, China; douhao@mail.nwpu.edu.cn (H.D.); YWG2017@163.com (W.Y.); dhzhang@nwpu.edu.cn (D.Z.); cowboyonm4rs@gmail.com (J.L.); 2016301091@mail.nwpu.edu.cn (Z.M.); 2 Department of Mechanical Engineering, University of Wisconsin-Madison, Madison, WI 53706, USA

**Keywords:** 3D printing, continuous carbon fiber, printing parameters, fiber-matrix interface, relative fiber content, tensile mechanical properties

## Abstract

Three-dimensional (3D) printing continuous carbon fiber-reinforced polylactic acid (PLA) composites offer excellent tensile mechanical properties. The present study aimed to research the effect of process parameters on the tensile mechanical properties of 3D printing composite specimens through a series of mechanical experiments. The main printing parameters, including layer height, extrusion width, printing temperature, and printing speed are changed to manufacture specimens based on the modified fused filament fabrication 3D printer, and the tensile mechanical properties of 3D printing continuous carbon fiber-reinforced PLA composites are presented. By comparing the outcomes of experiments, the results show that relative fiber content has a significant impact on mechanical properties and the ratio of carbon fibers in composites is influenced by layer height and extrusion width. The tensile mechanical properties of continuous carbon fiber-reinforced composites gradually decrease with an increase of layer height and extrusion width. In addition, printing temperature and speed also affect the fiber matrix interface, i.e., tensile mechanical properties increase as the printing temperature rises, while the tensile mechanical properties decrease when the printing speed increases. Furthermore, the strengthening mechanism on the tensile mechanical properties is that external loads subjected to the components can be transferred to the carbon fibers through the fiber-matrix interface. Additionally, SEM images suggest that the main weakness of continuous carbon fiber-reinforced 3D printing composites exists in the fiber-matrix interface, and the main failure is the pull-out of the fiber caused by the interface destruction.

## 1. Introduction

Three-dimensional (3D) printing (additive manufacturing) is a new manufacturing process, which uses a three-dimensional model (CAD model) to create a desired object by adding materials in layers. At present, 3D printing uses a variety of materials, including acrylonitrile-butadiene-styrene (ABS) [1,2,3] polylactic acid (PLA) [1,3,4], polyamide (PA) [5], and other thermoplastics, as well as thermosetting plastics such as epoxy resins. Although 3D printing technology has many advantages that are unmatched by other manufacturing processes, most 3D printing plastic structural parts are still in the stage of conceptual prototypes because the strength and performance of 3D printing plastic structural parts are much lower than those of functional components. It cannot be used as an actual function or bearing member.

Many studies have improved the mechanical properties of 3D printing parts by adding reinforcing materials such as fibers or particles. This method has been widely used in processes for enhancing the strength of conventional composites by forming fiber-reinforced polymers (FRP) [6]. Zhong et al. achieved the strength of 3D printing parts by adding chopped short glass fibers in the printing of ABS polymers. They also found that short glass fibers and ABS polymers could be made into composite filaments by using extrusion equipment to achieve good 3D printing quality [7]. The voids in the composite and the interaction of the fibers with the matrix directly limited the maximum strength of the fiber-reinforced composite [8,9,10]. Some studies have used expanded microspheres [9] or flaky fiber/fiber particles [10] instead of short fibers to reduce porosity in the composite while increasing fiber–matrix interaction.

Most of the research has focused on the development of short fiber-reinforced materials [11,12,13,14]. Although short fiber-reinforced composites have better mechanical properties than unreinforced composites, they have a substantial gap in mechanical properties as compared with continuous fiber-reinforced composites [15,16,17,18,19].

Li [15] proposed a continuous fiber-reinforced 3D printing method and a matching path control method, and by modifying the existing printer nozzle, the printing of continuous carbon fiber-reinforced PLA standard parts was realized. The mechanical properties and dynamic thermodynamic properties of continuous carbon fiber-reinforced composites were obtained by tensile tests, three-point bending tests, and dynamic mechanical experiments. Because the poor interface between carbon fiber and PLA seriously affects the mechanical properties of the composites [16], a carbon fiber surface preparation method was proposed, and the carbon fiber was modified by PLA dispersion slurry. The mechanical properties of the ordinary PLA, fiber-reinforced PLA, and modified fiber-reinforced PLA were compared and the experimental results showed that the tensile strength of carbon fiber-reinforced PLA was much higher than that of the ordinary PLA, and also the modification of carbon fiber improved the bending strength of the carbon fiber-reinforced PLA.

F. Van Der Klift [20] studied carbon fiber-reinforced Nylon^®^ composites with different fiber contents and predicted the elastic modulus of the samples using the composite volume-average method. The experimental results showed that carbon fiber could significantly improve the mechanical properties of composites. The elastic modulus of the low fiber content sample was in good agreement with the predicted results. For the high fiber content sample, the experimental results deviated significantly from the predicted results, because as the fiber content increased, the porosity of the sample also increased, which had a great effect on the predicted results.

Melenka et al. [21] evaluated the tensile mechanical properties of a material by evaluating Kevlar fiber-reinforced Nylon^®^ composites. The experimental results showed that the tensile strength and stiffness of the composites were significantly improved under the reinforcement of a high content of fibers. Meanwhile, the volume average method was used to predict the elastic parameters of fiber-reinforced 3D printing samples, with good prediction results for samples with high fiber content.

Dickson [22] compared the tensile mechanical properties and bending mechanical properties of carbon fiber, glass fiber, and Kevlar fiber-reinforced Nylon^®^ composites, and evaluated the effects of fiber content, fiber arrangement, and fiber direction on the mechanical properties of the samples. The results showed that carbon fiber had the strongest reinforcing effect, and Kevlar fiber had the smallest effect. As the fiber content increased, the mechanical properties of the sample gradually increased, but if the fiber content exceeded a certain ratio, the mechanical properties of the sample decreased due to an increase of porosity and the change of bonding properties between the fiber and the substrate.

Sanei [23] reviewed 3D printing of chopped and continuous carbon fiber composites to provide a reference for state-of-the-art efforts, exiting limitations, and new frontiers. The review showed that the choice of process parameters could lead to significant differences using the same material and printer. In detail, with different 3D printing process parameters for fiber-reinforced composites, such as infill pattern and density, fiber orientation, volume fraction, and stacking sequence, build orientation, specimens design and tabs, start/end point of fibers, there were significant differences in mechanical behavior.

Werken [24] examined the work performed in this fast-growing area. Specifically, the effects of fiber reinforcement on the structure and mechanical properties of 3D printing parts were investigated within the body of literature. Upper bounds for tensile properties of carbon fiber composites were theoretically evaluated and compared with experimentally measured values. Moreover, current and potential applications of additively manufactured carbon fiber composites in the context of desktop 3D printing and big area additive manufacturing were discussed.

The purpose of this study is to determine the relationship between the tensile mechanical properties of continuous carbon fiber-reinforced composites and printing parameters by using a modified Fused Deposition Molding (FDM) 3D printer. The main printing parameters researched are printing layer height, extrusion width, printing temperature, and printing speed. According to the ASTM International Standard, the tensile mechanical properties of continuous carbon fiber-reinforced 3D printing composites are experimentally characterized.

In order to further study the strengthening mechanism of the above printing parameters on the tensile mechanical properties, the microstructures of the specimens’ cross sections are observed by using field emission scanning electron microscopy (FESEM), and the relative fiber content and fiber-matrix bonding interface strength are analyzed, then, the failure form of the specimens in the tensile test are summarized.

## 2. Continuous Carbon Fiber-Reinforced Composites Preparation

### 2.1. Experimental Materials: Polylactic Acid (PLA) and Carbon Fiber

**Figure 1:**
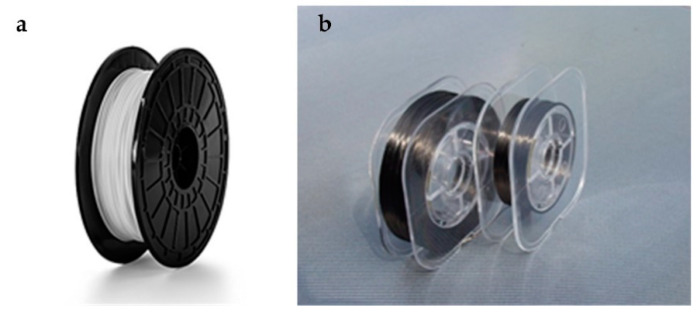
Kossel 3D Printer. (**a**) FlashForge polylactic acid (PLA) 3D filament; (**b**) HTA40 1K carbon fiber.

In this study, PLA is used as the base material. PLA is a biodegradable and thermoplastic plastic, which can be extracted from renewable resources such as corn and sweet potato, and is now widely used in 3D printing, food, and medical treatments. As shown in Figure 1, the PLA used in this paper is produced by FlashForge (Hangzhou, Zhejiang, China).

The weight of a single coil is 1 kg, and the diameter of PLA wire is 1.75 mm ± 0.02 mm. Carbon fiber as the reinforcing phase, is a light engineering material with high strength, high stiffness, low coefficient of thermal expansion, high temperature resistance, and excellent chemical resistance. These properties have resulted in carbon fiber being widely used in aviation, architecture, military, and racing fields. As shown in Figure 1, the carbon fiber used in this paper is 1K carbon fiber HTA 40 (HTA 40, Toho Tenax Co., Ltd., Tokyo, Japan). As one of Tenax series carbon fibers, HTA 40 is suitable for various processing technologies. The adhesion strength between carbon fiber and resin can be improved by surface treatment, and the fiber can be protected with good penetration rate and processability.

Before being used, all materials were sealed in a box, together with desiccant, to prevent the material from absorbing moisture, which could result in performance degradation. The mechanical properties are shown in Table 1.

### 2.2. Continuous Carbon Fiber 3D Printer Modification and Experimental Equipment

**Figure 2:**
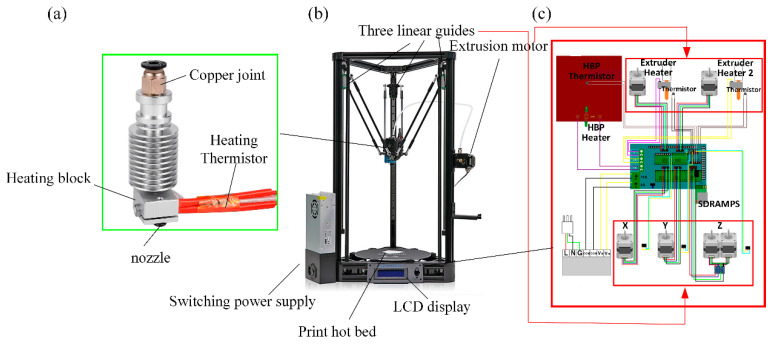
Kossel 3D Printer. (**a**) 3D printer head; (**b**) 3D printer overall structure; (**c**) Printer control board.

This study used a desktop-level RepRap Kossel 3D (Seattle, USA) printer as a prototype and some transformations were done accordingly. The RepRap Kossel 3D printer is an open source printer released by Johann [25,26] under the General Public License (GPL). The composition of the Kossel 3D printer is shown in Figure 2.

The Delta parallel motion mechanism is driven by three independent stepping motors with an accuracy of ±0.1 mm. The motion solution was used to convert the XYZ three-coordinate motion of the print head into a linear slide.

**Figure 3:**
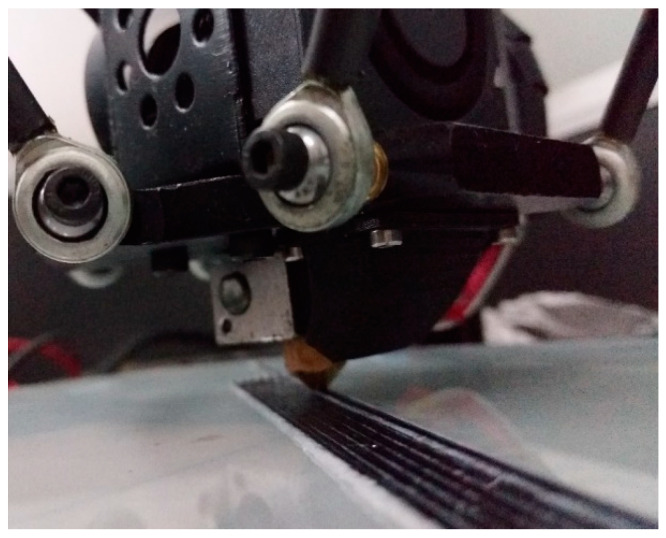
Continuous fiber-reinforced composite printing process.

The modification of the 3D printer was mainly concentrated at several positions such as nozzles, hoses, and printer fans. At the hot end of the printer, the PLA was heated to the glass transition temperature and became semi-liquid, at which point the carbon fiber was impregnated with the semi-liquid thermoplastic substrate. Under internal pressure, the semi-liquid thermoplastic and continuous carbon fiber were extruded from the nozzle and adhered to the printing platform. Since the average width of the carbon fibers was 0.9 mm, it was necessary to use a printing nozzle with a nozzle diameter of 1.5 mm. The printing process is as shown in Figure 3.

**Figure 4:**
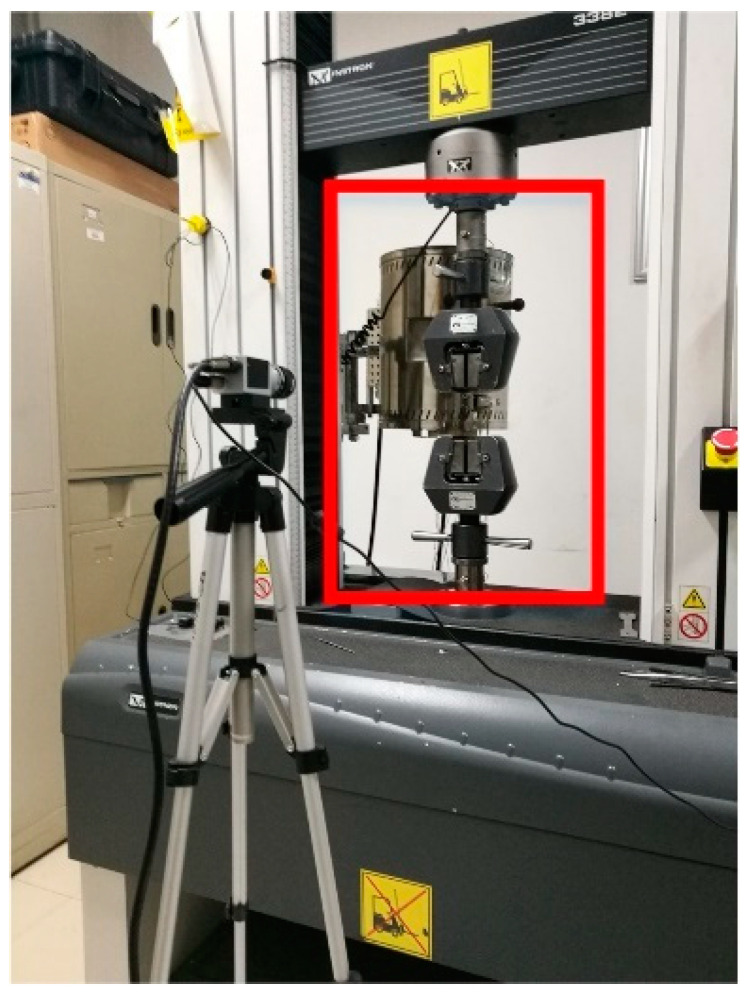
Experimental equipment (INSTRON 3382, INSTRON).

In order to evaluate the effect of process parameters on the tensile mechanical properties, as shown in Figure 4, the tensile mechanical properties of the specimens were tested using a 100 kN universal testing machine (INSTRON 3382, INSTRON, Boston, USA). Force and displacement data were collected by the sensor, and the nominal stress-strain curve of the sample was automatically calculated. The experimental rate of all samples was quasi-static, which was 1 mm/min.

### 2.3. Experimental Design and Print Parameter Selection

In this study, the tensile mechanical properties of the samples were tested using ASTM D3039-07 (Standard Test Method for Tensile Properties of Resin-Based Composites). The 3240 epoxy resin plates, with a thickness of 1.5 mm, were used at both ends of the sample to make the clamping portion. The clip and the sample were bonded with a cyanoacrylate adhesive (Ergo 5800), and after 24 h at room temperature, the adhesive was completely cured.

As for the production of tensile specimens, the minimum length of the tensile specimen can be calculated according to the following formula, Equation (1):

(1) $$L_{c}=L_{g}+L_{e}+2\times W$$

where *L_c_* is the minimum length of the sample/mm, *L_g_* is the length of the clamping portion of the sample/mm, *L_e_* is the length of the strain extensometer during the tensile test/mm, and *W* is the width of the sample/mm.

**Figure 5:**
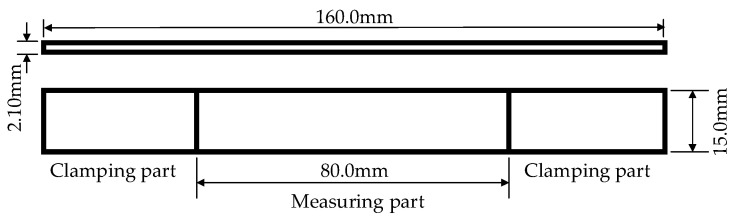
Continuous fiber-reinforced 3D printing tensile specimen.

In this study, according to the above formula and the recommended values in the standard, taking into account the maximum print size of the 3D printer, the dimensions of the designed tensile specimen were as follows: length of 160 mm, width of 15 mm, and height is of 2.1 mm, as shown in Figure 5.

The sample designed in SolidWorks was imported into the slicing software to generate the G-Code file required for 3D printing. Repetier-Host was used to control the 3D printer to complete the printing of the continuous carbon fiber-reinforced tensile sample.

In the experiment, there was a wedge clamp at each end of the test machine to fix the sample. In order to ensure that the force of the specimen was completely parallel to the length direction, sufficient lateral preload was applied to prevent the specimen from slipping and micro-rotation. Therefore, it was necessary to use clips. On the one hand, the clips provided sufficient pretightening force to prevent the specimen from slipping. On the other hand, the clips prevented excessive preload from damaging the surface of the specimen, which could result in abnormal fracture. According to the standard, the bevel of the clip is 5°~90° and the optimum angle is 7°~10°. Therefore, in this study, the angle of the clip was 10° and the thickness of the clip was 1.5 mm. The minimum length of the clip can be estimated according to the following formula, Equation (2):

(2) $$L_{t}=\frac{F^{tu}h}{2F^{su}}$$

where *L_t_* is the shortest length of the clip/mm, *F^tu^* is the tensile strength of the sample material/MPa, and *h* is the sample thickness/mm.

**Figure 6:**
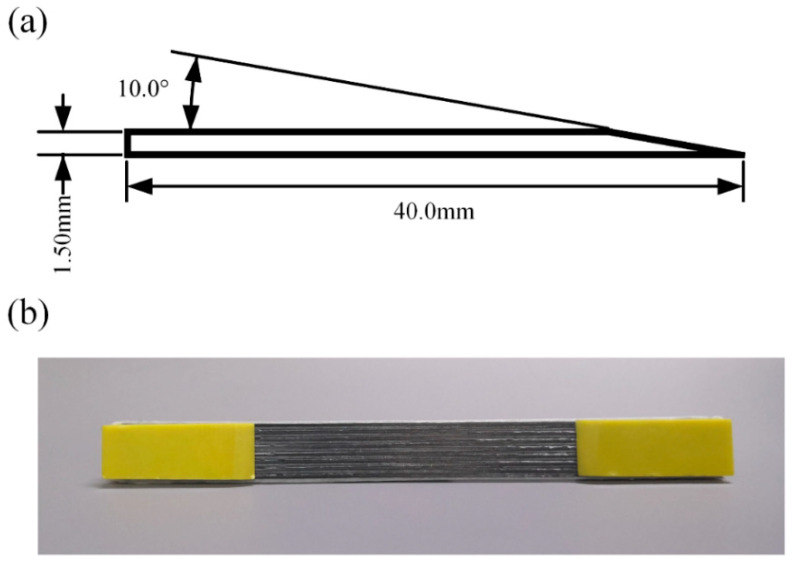
Clip size and final specimen. (**a**) Dimensions of clips; (**b**) Continuous carbon fiber-reinforced 3D printing tensile specimen.

According to the previous experiments, the length of the clip was chosen as 40 mm, and its size is shown in Figure 6a.

Cyanoacrylate adhesive (Ergo 5800) was used to bond the processed sample and clip. The finished sample is shown in Figure 6b.

**Figure 7:**
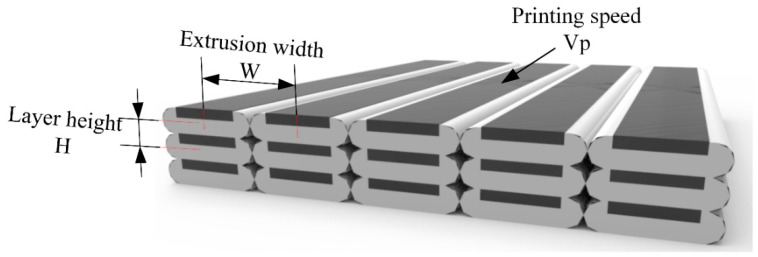
Sketch of 3D printing parameters.

During a 3D printing process, there are many parameters that affect the final mechanical properties of continuous carbon fiber-reinforced 3D printing parts. The most important parameters are layer height, printing speed, extrusion width, printing pitch, printing temperature, and printing direction. In order to study the influence of parameters on the mechanical properties of continuous carbon fiber-reinforced 3D printing parts, we considered that related research on the influence of printing direction on the quality of printing parts had matured, and that there was a certain coupling relationship between extrusion width and printing pitch, i.e. the variation range of the printing pitch is very small when the width is constant. Therefore, in this study, we mainly researched the influence of layer height, extrusion width, printing temperature, and printing speed on the tensile mechanical properties, and experiments were carried out by means of control variables. The main printing parameters are shown in Table 2 and the experimental design is shown in Figure 7.

During the process of printing tensile specimens, other fixed parameters are shown in Table 3.

## 3. Tensile Mechanical Properties Analysis of Continuous Carbon Fiber-Reinforced 3D Printing Standard Parts

In order to obtain more comprehensive comparison results, in this study, we used the ASTM D3039-07 (Standard Test Method for Tensile Properties of Resin-Based Composites) to compare the tensile mechanical properties of the samples. The sample size was specified by the standard as mentioned above, and the experiments were carried out at a quasi-static rate of 1 mm/min. 

### 3.1. Effect of 3D Printing Parameters on Tensile Mechanical Properties

#### 3.1.1. Effect of Printing Layer Height on Tensile Mechanical Properties 

For traditional 3D printing technology, layer height is a very important printing parameter as the forming characteristic of FDM is stacking layer by layer, so the layer height directly affects the printing accuracy, printing efficiency, and mechanical properties of FDM 3D printing. As shown in Figure 7, as the layer height decreased, higher accuracy of 3D printing in the Z direction could be obtained, more layers needed to be printed, and the printing time increased accordingly.

In continuous carbon fiber-reinforced 3D printing, the layer height has a more profound impact. Because of the commercial 1K fiber bundle used in this paper, the total amount of carbon fiber on a single printing path could not be changed. Therefore, relative fiber content was adjusted by changing the proportion of matrix material. From Figure 7, the smaller the layer height, the higher the relative content of carbon fiber.

**Figure 8:**
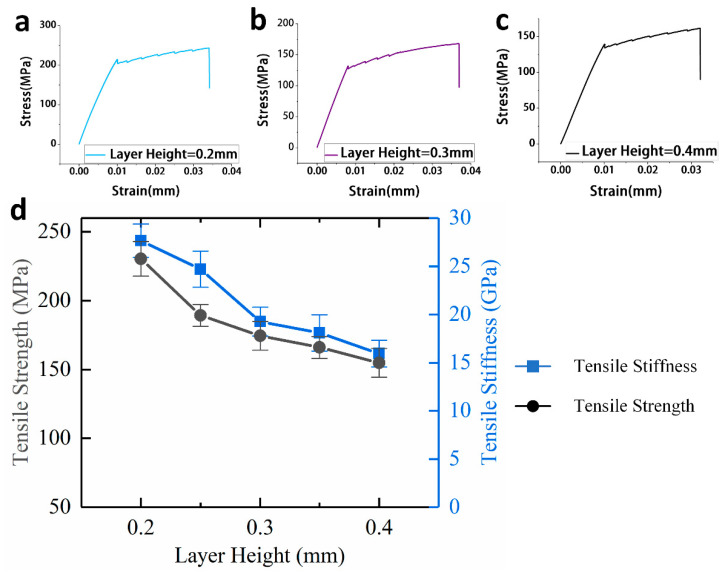
(**a**) Tensile stress-strain curve of specimens while the layer height is 0.2 mm; (**b**) Tensile stress-strain curve of specimens while the layer height is 0.3 mm; (**c**) Tensile stress-strain curve of specimens while the layer height is 0.4 mm; (**d**) Effect of layer height on tensile mechanical properties of continuous carbon fiber-reinforced 3D printing.

As shown in Figure 8d, when the layer height is increased from 0.2 mm to 0.4 mm, the tensile strength and tensile stiffness of the continuous carbon fiber-reinforced composites gradually decrease.

When the layer height is 0.2 mm, the continuous carbon fiber-reinforced composites have the highest tensile mechanical properties, and the tensile strength and tensile stiffness are 243.53 MPa and 25.77 GPa, respectively. When the layer height is 0.4 mm, the continuous carbon fiber-reinforced composites have the lowest tensile mechanical properties, and the tensile strength and tensile stiffness are 161.61 MPa and 14.99 GPa.

#### 3.1.2. Effect of Extrusion Width on Tensile Mechanical Properties

Extrusion width refers to the distance between the centerlines of two adjacent print paths, and, to a certain extent, extrusion width will affect the porosity of 3D printing. For continuous carbon fiber-reinforced 3D printing, the effect of extrusion width on the tensile mechanical properties is similar to that of printing layer height. When the extrusion width becomes smaller, more printing paths are needed for a single layer, so the relative fiber content in the single layer increases.

**Figure 9:**
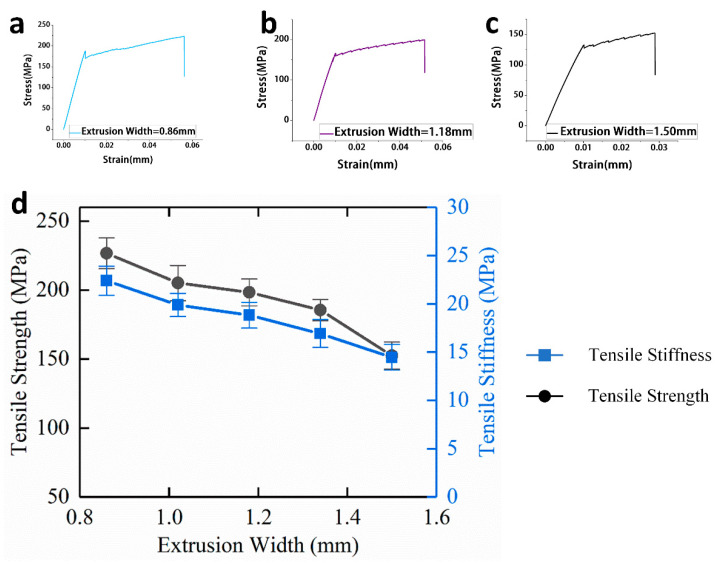
(**a**) Tensile stress-strain curve of specimens while the extrusion width is 0.86 mm; (**b**) Tensile stress-strain curve of specimens while the extrusion width is 1.18 mm; (**c**) Tensile stress-strain curve of specimens while the extrusion width is 1.50 mm; (**d**) Effect of extrusion width on tensile mechanical properties of continuous carbon-reinforced 3D printing.

As shown in Figure 9d, when the extrusion width is increased from 0.86 to 1.5 mm, the tensile mechanical properties of the continuous carbon fiber-reinforced composites are gradually lowered.

When the extrusion width is 0.86 mm, the tensile strength and tensile stiffness reach a maximum of 226.60 MPa and 22.38 GPa. When the extrusion width is 1.5 mm, the continuous carbon fiber-reinforced composites have the lowest tensile mechanical properties, and the tensile strength and stiffness are 152.41 MPa and 14.46 GPa, respectively.

#### 3.1.3. Effect of Printing Temperature on Tensile Mechanical Properties

As a key parameter of 3D printing, printing temperature refers to the temperature at the hot end nozzle of the printer during printing, and the printing temperature mainly affects the strength of the bonding interface of the samples. For continuous carbon fiber-reinforced 3D printing, because the impregnation mechanism of the fiber and matrix is in situ impregnation, the printing temperature affects the bonding interface between the fiber and the matrix.

**Figure 10:**
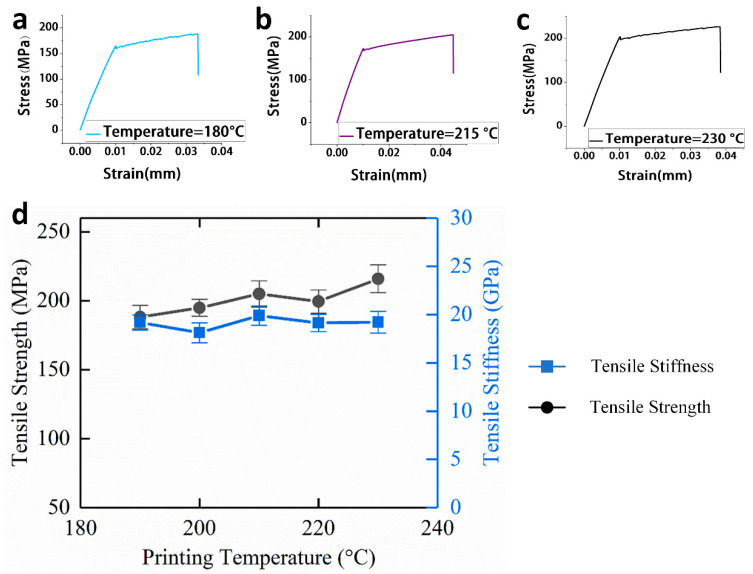
(**a**) Tensile stress-strain curve of specimens while the printing temperature is 180 °C; (**b**) Tensile stress-strain curve of specimens while the printing temperature is 215 °C; (**c**) Tensile stress-strain curve of specimens while the printing temperature is 230 °C; (**d**) Effect of printing temperature on tensile mechanical properties of continuous carbon fiber-reinforced 3D printing.

As shown in Figure 10d, the tensile strength increases when the printing temperature is raised from 180 to 230 °C, which is determined by the matrix material, but the tensile stiffness does not change significantly when the printing temperature is 230 °C as compared with a lower temperature.

The continuous carbon fiber-reinforced composites have a tensile strength of 210.96 MPa and a tensile modulus of 19.23 GPa. This is because the tensile strength of the continuous carbon fiber-reinforced composites are affected by the fiber-matrix bonding interface, and the tensile stiffness of the composites mainly depends on the tensile stiffness of the continuous carbon fiber, therefore, when the temperature rises, the fiber-matrix bonding interface is gradually enhanced, and the tensile strength is also increased, but the tensile stiffness remains substantially unchanged.

#### 3.1.4. Effect of Printing Speed on Tensile Mechanical Properties

Printing speed refers to the movement speed of a 3D printer nozzle during printing. For traditional FDM printing, the printing speed mainly affects the forming accuracy and efficiency. However, for continuous carbon fiber-reinforced 3D printing, since the printing speed determines the impregnation time of fiber and matrix at the hot end of the printer, the printing speed also affects the fiber matrix interface of the composites. Continuous carbon fiber-reinforced 3D printing has certain requirements for printing speed. When the extrusion speed is too high, continuous carbon fiber is easy to fracture, whereas, when the printing speed is too slow, the printing efficiency is deficient, therefore, the chosen printing speed ranges from 50 to 400 mm/min.

**Figure 11:**
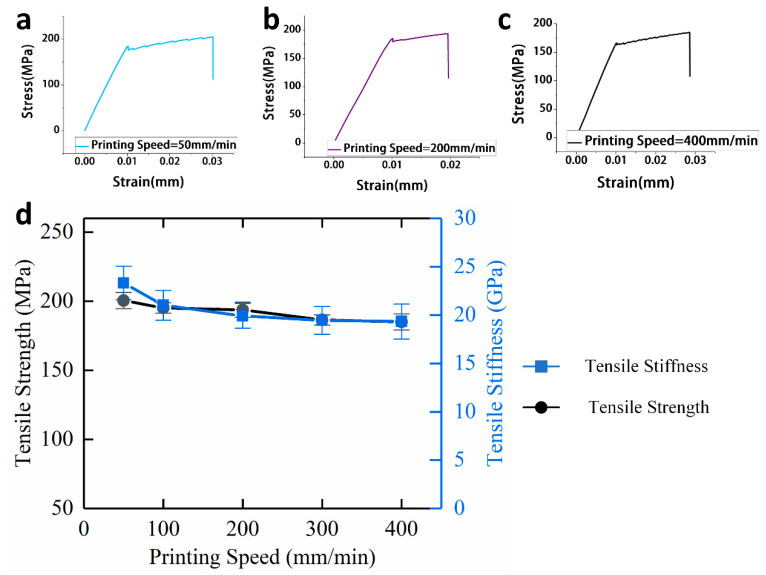
(**a**) Tensile stress-strain curve of specimens while the printing speed is 50 mm/min; (**b**) Tensile stress-strain curve of specimens while the printing speed is 200 mm/min; (**c**) Tensile stress-strain curve of specimens while the printing speed is 400 mm/min; (**d**) Effect of printing speed on tensile mechanical properties of continuous carbon fiber-reinforced 3D printing.

As shown in Figure 11d, when the printing speed is increased from 50 to 400 mm/min, the tensile strength and the tensile stiffness gradually decline.

At a printing speed of 50 mm/min, the tensile strength and tensile stiffness of the specimens are the highest, at 200.43 MPa and 23.31 GPa, respectively, whereas, at a printing speed of 400 mm/min, the tensile strength and tensile stiffness are 185 MPa and 19.33 GPa, respectively. Therefore, continuous carbon fiber-reinforced 3D printing composites have better tensile mechanical properties when the printing speed is lower, which is mainly due to two reasons. On the one hand, there is a longer immersion time between continuous carbon fiber and basal body before being extruded, which results in a stronger bonding interface between the carbon fibers and the matrix, and thus the tensile strength is increased. On the other hand, there are some associations between the printing speed and the extrusion speed. The extrusion speed decreases when the printing speed is lower, which means continuous carbon fibers receive less compressive force in the nozzle, therefore, continuous carbon fibers maintain good surface topography and orientation uniformity, which results in better tensile stiffness for continuous carbon fiber-reinforced 3D printing composites.

#### 3.1.5. Effect of Relative Fiber Content on Tensile Mechanical Properties

For continuous carbon fiber-reinforced 3D printing composites, the relative fiber content is affected by two important parameters, printing layer height and the extrusion width. Since the carbon fiber and the matrix material are simultaneously extruded from the nozzle during the printing process, the total length of the printing path is substantially equal to the carbon fiber length in the continuous carbon fiber-reinforced composites, so the relative fiber content can be calculated according to the following formula, Equation (3):

(3) $$\omega_{c}=\frac{\rho_{c}L}{W_{e}H_{l}L\rho_{s}}=\frac{\rho_{c}}{WH_{l}\rho_{s}}$$

where *ρ_c_* is the linear density of carbon fiber/(kg/m), *ρ_s_* is the volume density of matrix materials/(kg/m^3^), *L* is the total print path length/(mm), *W_e_* is the extrusion width/(mm), and *H_l_* is the printing layer height/mm.

**Figure 12:**
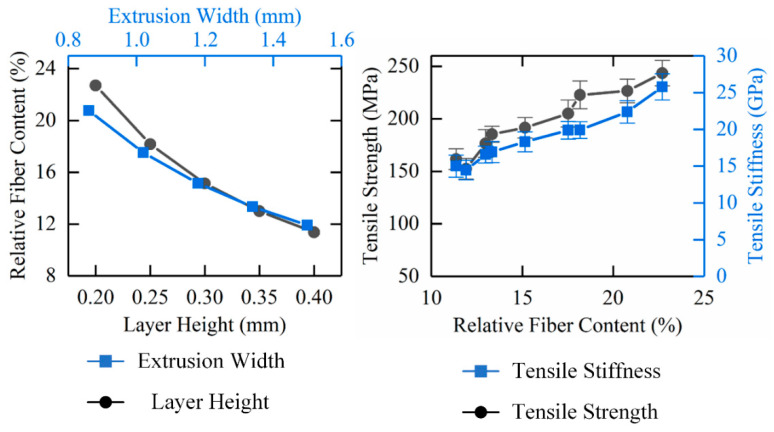
Effect of relative fiber content on tensile mechanical properties of continuous carbon fiber-reinforced 3D printing.

As shown in Figure 12, when the layer height or extrusion width increases, the relative fiber content in the continuous carbon fiber-reinforced composite decreases gradually.

In the meantime, the tensile mechanical properties of the composites will gradually rise with an increase of carbon fiber content. The maximum relative fiber content is 22.7%, when the printing layer height is 0.2 mm and the extrusion width is 1.18 mm, while the tensile strength is 243.53 MPa, and the tensile stiffness is 25.77 GPa.

Compared with the influence of relative fiber content on the tensile mechanical properties of continuous carbon fiber-reinforced composites, the printing temperature and printing speed have much less impact, mainly because the two parameters affect the fiber-matrix bonding interface of the 3D printing composites, however, the tensile mechanical properties of continuous carbon fiber-reinforced 3D printing composites are mainly derived from the reinforcing phase, continuous carbon fiber. As a result, for continuous carbon fiber-reinforced 3D printing composites, the printing layer height and extrusion width are the two most important process parameters that affect the carbon fiber content of the composites, and thus the tensile mechanical properties of the composites will be influenced.

### 3.2. Enhancement Mechanism and Fracture form Analysis

**Figure 13:**
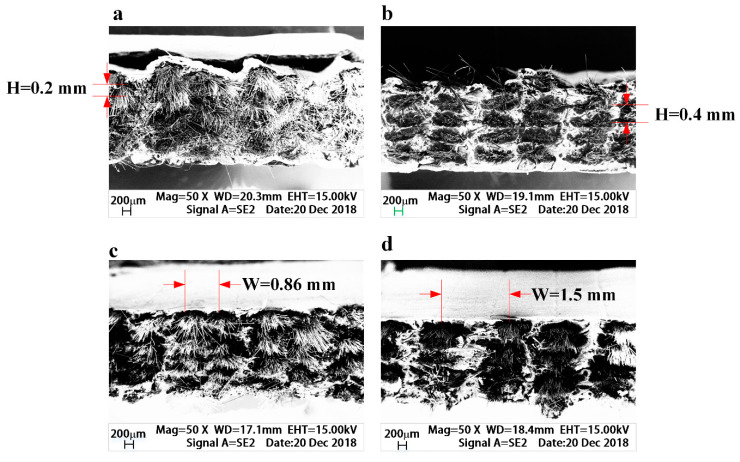
Fracture cross section of tensile specimens with different relative fiber content. (**a**) H = 0.2 mm; (**b**) H = 0.4 mm; (**c**) W = 0.86 mm; (**d**) W = 1.5 mm.

Figure 13a,b shows the fracture sections of continuous carbon fiber-reinforced 3D printing composites tensile samples having the printing layer height of 0.2 mm and 0.4 mm, respectively, which are obtained by using field emission scanning electron microscopy (FESEM)

As shown in the SEM images, as the height of the printing layer rises, the space of the carbon fibers between any two layers increases, therefore, when the printing layer height is very small, the continuous carbon fiber-reinforced 3D printing composites have higher carbon content. Similarly, as shown in Figure 13c,d, the fracture cross section of the reinforced 3D printing composites tensile samples having an extrusion width of 0.86 and 1.5 mm, respectively. When the extrusion width is 0.86 mm, the distance between an adjacent two rows of carbon fibers is relatively close, while extrusion width is 1.50 mm, the distance is longer, that is, when the extrusion width is small, more reinforced continuous carbon fiber can be contained.

**Figure 14:**
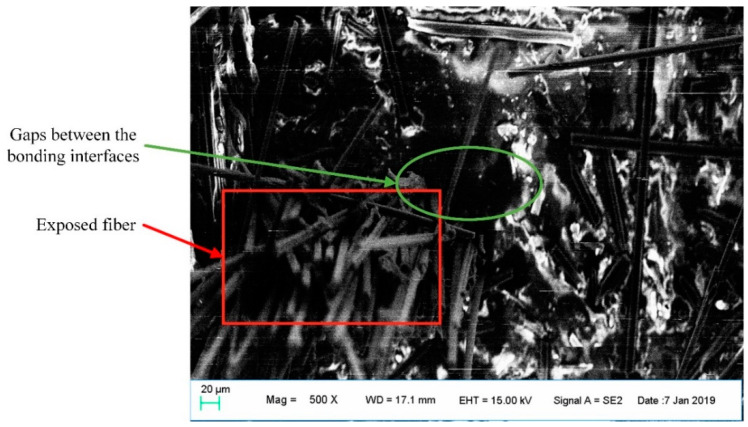
Fiber-matrix bonding interface of continuous carbon fiber-reinforced 3D printing parts.

Figure 14 shows a carbon fiber-matrix bonding interface, through which the tensile stress loaded on the PLA substrate can be transferred to the continuous carbon fiber, and therefore the carbon fiber can enhance the tensile mechanical properties of the composites.

However, the gaps between the bonding interfaces indicate that the carbon fibers cannot be completely fused with the PLA matrix, therefore, the strength at the carbon fiber-matrix bonding interface is not ideal enough. This incomplete impregnation causes the composites to be unable to fully utilize the mechanical reinforcement of carbon fiber. As a result, the main failure mode of the continuous carbon fiber-reinforced composites is the damage of carbon fiber-matrix bonding interface, and therefore the carbon fiber is extracted from the matrix instead of completely breaking during the experiments.

## 4. Conclusions

The tensile mechanical properties of continuous carbon fiber-reinforced 3D printing composites is affected by various process parameters. The printing layer height and extrusion width affect the proportion of carbon fiber inside the composites. When the printing layer height is increased from 0.2 to 0.4 mm and the extrusion width is increased from 0.86 to 1.5 mm, the tensile mechanical properties decrease, because the relative fiber content has a significant impact on the tensile mechanical properties of the composites. The printing temperature and printing speed can affect the fiber-matrix bonding interface. When the printing temperature is raised from 190 to 230 °C and the printing speed is increased from 50 to 400 mm/min, the tensile mechanical properties decrease gradually. Compared with the relative fiber content, the fiber-matrix interface has little influence on the tensile mechanical properties. The main weakness of continuous carbon fiber-reinforced 3D printing composites lies in the fiber-matrix interface, and the main failure mode of continuous carbon fiber is fiber pull-out caused by interface failure, which means that future research should focus on strengthening the interface between the carbon fiber and matrix.

## Figures and Tables

**Table 1:** Mechanical properties of HTA 40 and PLA.

Material	Tensile Strength	Tensile Modulus	Breaking Elongation Rate	Density
HTA 40	4100 MPa	240 GPa	1.7%	$1.77\;g/{\mathrm{cm}}^{3}$
PLA filament	62.63 MPa	3.2 GPa	4.43%	$1.24\;g/{\mathrm{cm}}^{3}$

**Table 2:** Parameters of the carbon fiber-reinforced 3D printing tensile specimen.

Printing Parameters	Parameter Range	Other Fixed Parameters
Printing layer height (*H*/mm)	0.20, 0.25, 0.30, 0.35, 0.40	W 1.18 T 210 V 100
Extrusion width (*W*/mm)	0.86, 1.02, 1.18, 1.34, 1.50	H 0.3 T 210 V 100
Printing temperature (*T*/°C;)	190, 200, 210, 220, 230	H 0.3 W 1.18 V 100
printing speed (*V*/$\mathrm{mm}\cdot \min^{-1}$)	50, 100, 200, 300, 400	H 0.3 W 1.18 T 210

**Table 3:** Fixed parameters of carbon fiber-reinforced 3D printing tensile specimen.

Infill Density	Pattern	Bed Temperature	Floor & Roof	Shell	Brim
100%	Zigzag	50 °C	No	No	1
